# Supplementary material for: Potential Clinically Relevant Effects of Sialylation on Human Serum AAG-Drug Interactions Assessed by Isothermal Titration Calorimetry: Insight into Pharmacoglycomics?
Source: Int J Mol Sci. 2023 May 9;24(10):8472. doi: 10.3390/ijms24108472 (PMC10218007; doi:10.3390/ijms24108472)
Supplement: Supplementary file 1 [file ijms-24-08472-s001.zip › ijms-2279270-supplementary.pdf]

Supporting Information

Potential Clinically Relevant Effects of Sialylation on Human Serum AAG-Drug Interactions Assessed by Isothermal Titration Calorimetry: Insight into Pharmacoglycomics?

Robert Kerep [1], Tino Šeba [1], Valentina Borko [1], Tin Weitner [1], Toma Keser [2], Gordan Lauc [2] and Mario Gabričević [1,*]

[1] Department of General and Inorganic Chemistry, Faculty of Pharmacy and Biochemistry, University of Zagreb, 10000 Zagreb, Croatia
[2] Department of Biochemistry and Molecular Biology, Faculty of Pharmacy and Biochemistry, University of Zagreb, 10000 Zagreb, Croatia
* Correspondence: mgabricevic@pharma.hr; Tel.: + 385-1-4818-307

Contents

1. Tables S1-S5 2
2. Figures S1-S6 5
3. References 9

1. Tables S1-S5

Table S1. Content of N-glycans in the native and desialylated AAG determined by UPLC-MS. The dominant fractions with content ≥ 5 % are bolded in red.

AAG+s	Structure*	Content %	AAG−s	Structure*	Content %
1	A2G2	0.25	1	A2G2	6.84
2	A2G2S1	2.17			
3	A3G3S1	1.3			
4	A2G2S2	0.5			
5	A2G2S2<br>A3G3S1	7.03	**2**	**A3G3**	**39.29**
**6**	**A3G3S2**	**11.27**			
7	A3G3S2	7.73			
8	A3F1G3S2	4.49	3	A3F1G3	12.38
9	A3G3S3	3.23	4	A3G3S1	8.72
**10**	**A3G3S3**	**24.55**			
11	A3G3S3	5.12			
12	A3F1G3S3	12.17	6	A3F1G3S1	5.21
13	A4G4S3	3.78	**5**	**A4G4**	**17.16**
14	A4G4S4	3.57			
15	A4G4S4<br>AFG4S3Lac	7.07	8	A4G4S1<br>A4G4Lac	5.12
16	AFG4S3Lac<br>A4F1G4S4	5.76	7	A4F1G4	5.29
**IS**		**313.11**	**IS**		**19.05**

*Structure abbreviations: all N-glycans have two core GlcNAcs; Ax, number of antenna (GlcNAc) on trimannosyl core; A2, biantennary with both GlcNAcs as β1,2-linked; A3, triantennary with a GlcNAc linked β1,2 to both mannose and the third GlcNAc linked β1,4 to the α1,3 linked mannose; A4, GlcNAcs linked as A3 with additional GlcNAc β1,6 linked to α1,6 mannose; Gx, number (x) of β1,4 linked galactose on antenna; F(x), number (x) of fucose linked α1,3 to antenna GlcNAc; Sx, number (x) of sialic acids linked to galactose; Lac(x), number (x) of lactosamine (Galβ1-4GlcNAc) extensions (1).

Table S2. The stability (association) constants, log K_A, for the drug complexes with AAG or AAG−s. The values are defined as log K_A = log ($1/K_D$) and signify drug concentrations at which half of available protein binding sites have been occupied. Higher values correspond to stronger binding.

Drug	AAG+s	AAG−s
	**log K_A***	**log K_A***
Lidocaine	5.03 ± 0.0440	5.28 ± 0.0770
Diltiazem	4.88 ± 0.0160	5.11 ± 0.0810
Warfarin	4.84 ± 0.0850	5.78 ± 0.0390
Clindamycin	4.51 ± 0.0340	4.48 ± 0.0440

*The uncertainties are given as standard deviation of the mean (N ≥ 2).

Table S3. Reference values for logarithms of association constants, log K_A, for the AAG-drug complex. The values are defined as log K_a = log ($1/K_D$); values of K_D taken in M. Higher values correspond to stronger binding.

**Drug**	**AAG+s**	**AAG−s**
	**log K_A**	**log K_A**
Lidocaine	5.18 (2)	ND
Diltiazem	ND*	ND
Warfarin	5.34 (3)	ND
Clindamycin	6.03 (4)	ND

*ND – not determined

Table S4. Acid dissociation constants (pK_a) for examined drugs.

**Drug**	**pK_a**
Lidocaine	7.93 (5)
Diltiazem	8.03 (5)
Clindamycin	7.79 (6)
Warfarin	4.99 (7)

Table S5. Estimation of the statistical significance for the influence of sialylation on the thermodynamic parameters for drug binding to AAG. Two-tailed *t*-test for independent samples assuming equal variances was used (ttest() function in Microsoft Excel).

Parameter	Sample	Student *t*-test		Parameter	Sample	Student *t*-test	
		p	sig.			*p*	sig.
K_D	AAG+s and clindamycin	0.608	no	$\Delta_r H°$	AAG+s and clindamycin	0.109	no
	AAG−s and clindamycin				AAG−s and clindamycin		
	AAG+s and diltiazem	0.0378	yes		AAG+s and diltiazem	0.964	no
	AAG−s and diltiazem				AAG−s and diltiazem		
	AAG+s and lidocaine	0.045	yes		AAG+s and lidocaine	0.336	no
	AAG−s and lidocaine				AAG−s and lidocaine		
	AAG+s and warfarin	0.0237	yes		AAG+s and warfarin	0.161	no
	AAG−s and warfarin				AAG−s and warfarin		
$-T\Delta_r S°$	AAG+s and clindamycin	0.131	no	$\Delta_r G°$	AAG+s and clindamycin	0.698	no
	AAG−s and clindamycin				AAG−s and clindamycin		
	AAG+s and diltiazem	0.752	no		AAG+s and diltiazem	0.0474	yes
	AAG−s and diltiazem				AAG−s and diltiazem		
	AAG+s and lidocaine	0.244	no		AAG+s and lidocaine	0.0532	no
	AAG−s and lidocaine				AAG−s and lidocaine		
	AAG+s and warfarin	0.0704	no		AAG+s and warfarin	0.00160	yes
	AAG−s and warfarin				AAG−s and warfarin		

2. Figures S1-S6

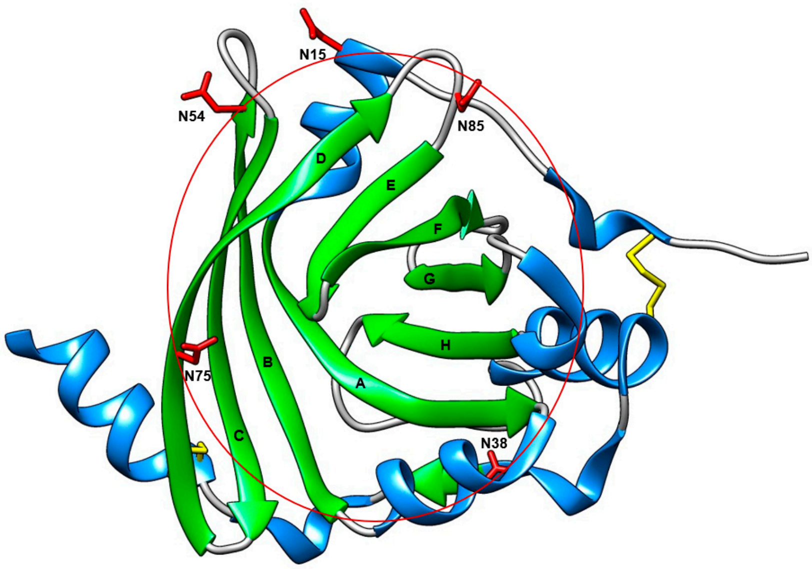

Figure S1. A ribbon representation of the structure for human deglycosylated AAG. Drawn according to PDB entry 3KQ0.pdb (8) which was created using the free UCSF Chimera program available online (https://www.rbvi.ucsf.edu/chimera). The deglycosylated AAG X–ray crystal structure consists of five N−linked glycosylation sites (red, Asn), α-helices (blue), β−strands (green), turns and loops (grey), two disulphide bonds connecting Cys residues 5–147 and 72–165 (yellow). The eight–stranded β–strand corresponds to β–sheets A–H and four regions: loop 1 (A/B), loop 2 (C/D), loop 3 (E/F) and loop 4 (G/H). The drug binding site is circled with a red line.

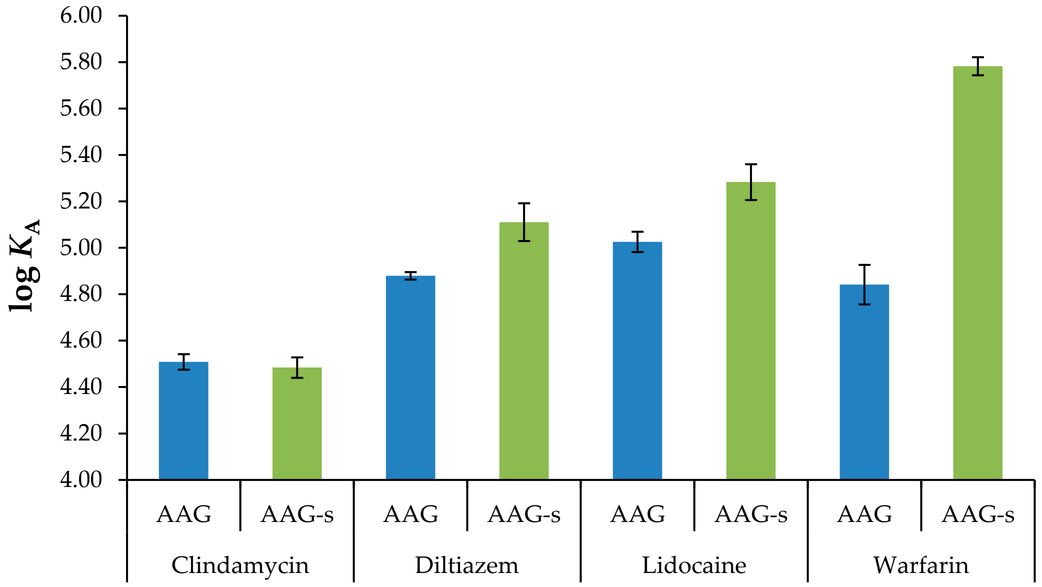

Figure S2. The stability (association) constants, log K_A, for the drug complexes with AAG+s or AAG−s. Higher values correspond to stronger binding. Error bars are standard deviations of measured constants.

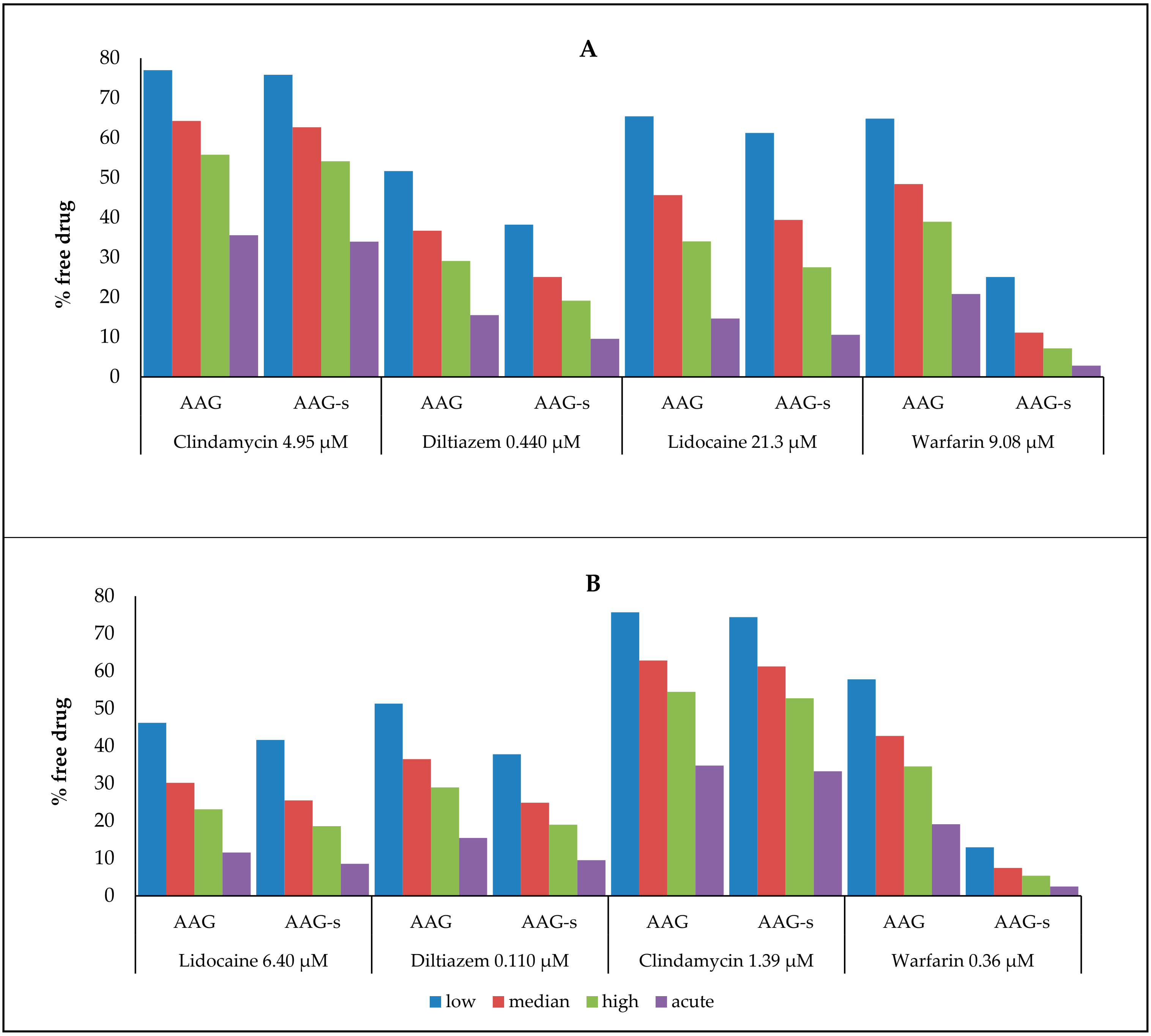

Figure S3. Percentage difference of free drug at (**A**) peak (C_{max}), and (**B**) trough therapeutic concentrations (C_{trough}), depending on the plasma concentrations (low, median, high and acute) of AAG+s or AAG−s.

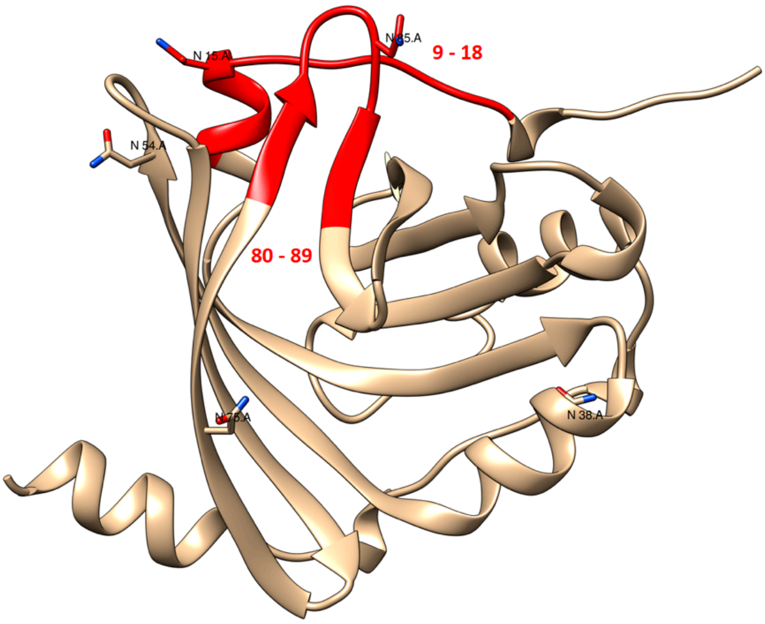

Figure S4. Peptide residues 9-18 and 80-89 (red) on the crystal structure of desialylated AAG that are structurally stabilized upon drug binding. The structure shows five N-linked residues on a single peptide chain (A). (https://www.rbvi.ucsf.edu/chimera).

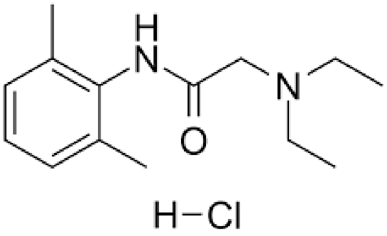

Lidocaine hydrochloride

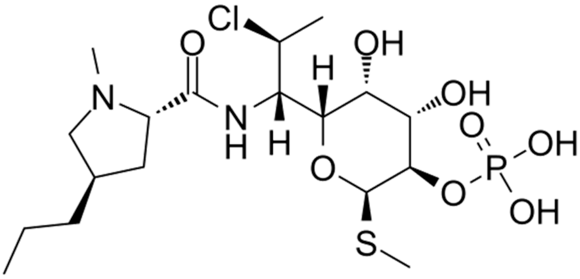

Clindamycin phosphate

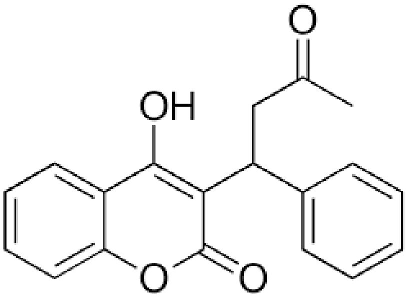

Warfarin

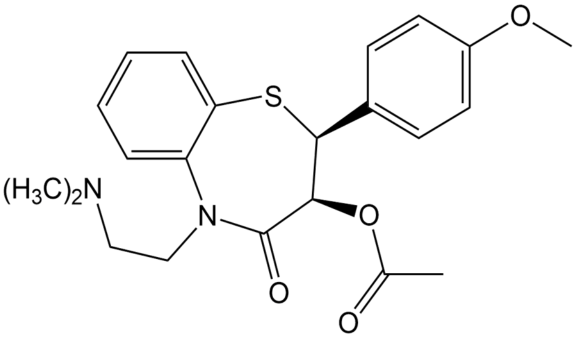

Diltiazem

Figure S5. Chemical structures of examined drugs.

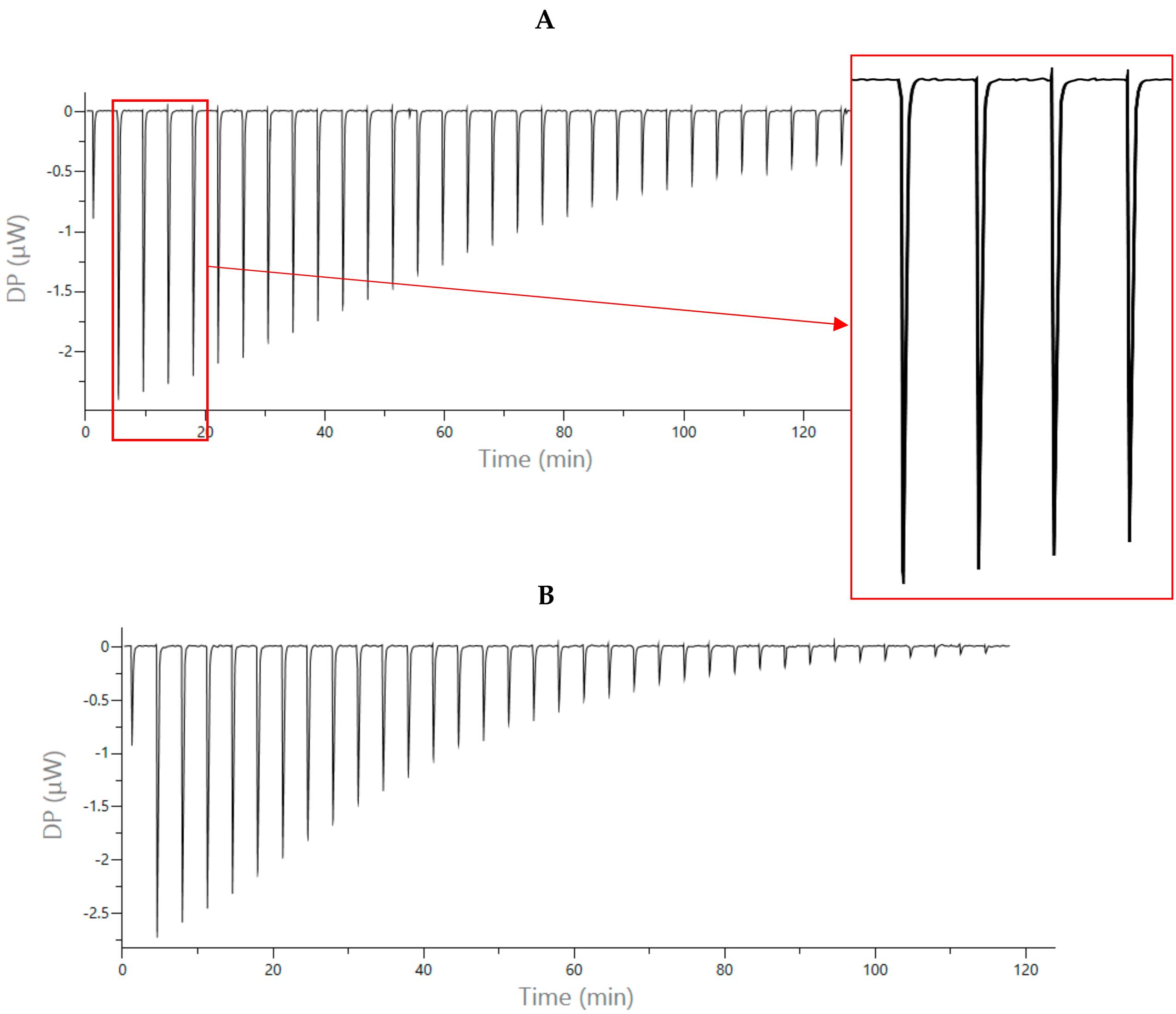

Figure S6. Microcalorimetric titrations for clindamycin with native (**A**) and desialylated (**B**) AAG. Figure shows the differential power in μW per injectant with subtracted baseline for better overview of peaks. Inset shows enlarged view of initial injections.

3. References

1. Saldova, R., Asadi Shehni, A., Haakensen, V. D., Steinfeld, I., Hilliard, M., Kifer, I., Helland, A., Yakhini, Z., Børresen-Dale, A.-L., and Rudd, P. M. Association of N-glycosylation with breast carcinoma and systemic features using high-resolution quantitative UPLC. *J. Proteome Res*. **2014**, *13*, 2314–2327
2. Soman, S., Yoo, M. J., Jang, Y. J., and Hage, D. S. Analysis of Lidocaine Interactions with Serum Proteins Using High-Performance Affinity Chromatography. *J. Chromatogr. B Analyt. Technol. Biomed. Life Sci*. **2010**, *878*, 705–708
3. Otagiri, M., Maruyama, T., Imai, T., Suenaga, A., and Imamura, Y. A comparative study of the interaction of warfarin with human alpha 1-acid glycoprotein and human albumin. *J Pharm Pharmacol*. **1987**, *39*, 416–420
4. Wulkersdorfer, B., Wicha, S.G., Kurdina, E., Carrion, Carrera, S.F., Matzneller, P., Al, Jalali, V., Vossen, M.G., Riesenhuber, S., Lackner, E., Dorn, C., Zeitlinger, M. Protein binding of clindamycin in vivo by means of intravascular microdialysis in healthy volunteers. *J. Antimicrob. Chemother*. **2021**, *76*, 2106-2113
5. Settimo, L., Bellman, K., and Knegtel, R. M. A. Comparison of the accuracy of experimental and predicted pKa values of basic and acidic compounds. *Pharm. Res*. **2014**, *31*, 1082–1095
6. Qiang, Z., and Adams, C. Potentiometric determination of acid dissociation constants (pKa) for human and veterinary antibiotics. *Water Res*. **2004**, *38*, 2874–2890
7. Nowak, P., Olechowska, P., Mitoraj, M., Woźniakiewicz, M., and Kościelniak, P. Determination of acid dissociation constants of warfarin and hydroxywarfarins by capillary electrophoresis. *J. Pharm. Biomed. Anal.* **2015**, *112*, 89–97
8. Schonfeld, D. L., Raimond, B. G. R., Uwe, M. and Skera, A. The 1.8-A crystal structure of alpha1-acid glycoprotein (Orosomucoid) solved by UV RIP reveals the broad drug-binding activity of this human plasma lipocalin. *J. Mol. Biol*. **2008**, *384*, 393-405
